# Probiotic *Bacillus* Attenuates Oxidative Stress- Induced Intestinal Injury via p38-Mediated Autophagy

**DOI:** 10.3389/fmicb.2019.02185

**Published:** 2019-09-30

**Authors:** Yanping Wu, Baikui Wang, Han Xu, Li Tang, Yali Li, Li Gong, Yang Wang, Weifen Li

**Affiliations:** ^1^Key Laboratory of Molecular Animal Nutrition of the Ministry of Education, Institute of Feed Science, College of Animal Sciences, Zhejiang University, Hangzhou, China; ^2^College of Animal Science and Technology, Zhejiang A & F University, Hangzhou, China; ^3^Animal Nutrition and Human Health Laboratory, School of Life Sciences, Hunan Normal University, Changsha, China

**Keywords:** oxidative stress, *Bacillus*, intestine, apoptosis, autophagy, p38 MAPK

## Abstract

Probiotics have been widely used in maintaining intestinal health and one of their benefits is to enhance host antioxidant capacity. However, the involved molecular mechanisms require further investigated. Autophagy is a self-protection process in response to diverse stresses. We hypothesized that probiotics could modulate intestinal autophagy to alleviate oxidative stress. Sprague-Dawley (SD) rats were orally administered *Bacillus* SC06 or SC08 daily for 24 days and thereafter received an intraperitoneal injection of diquat (DQ) to induce oxidative stress. We found that rats administered *Bacillus* SC06 showed more significant intestinal tissue repair and antioxidant properties than those administered SC08, which suggests a strain-specific effect of probiotics. Moreover, SC06 alleviated apoptosis by regulating the expression of Bcl2, Bax and cleaved caspase-3. Further investigations revealed that SC06 triggered autophagy, indicated by the upregulation of LC3 and Beclin1 and the degradation of p62 in rat jejunum and IEC-6 cells. Preincubation with autophagy inhibitor 3-methyladenine (3-MA) significantly aggravated reactive oxygen species (ROS) production and apoptotic cell formation. Furthermore, we demonstrated that p38 MAPK (mitogen-activated protein kinase), not AKT (alpha serine/threonine kinase)/mTOR (mammalian target of rapamycin), was involved in SC06-induced autophagy. Taken together, *Bacillus* SC06 can alleviate oxidative stress-induced disorders and apoptosis via p38-mediated autophagy. The above findings highlight a novel mechanism underlying the beneficial effects of probiotics as functional food and provide a new perspective on the prevention and treatment of oxidative damages.

## Introduction

Reactive oxygen species (ROS) are generated during cellular oxidative metabolism and normally function as redox messengers (Cadenas and Davies, 2000). At low levels, ROS can be eliminated by antioxidant systems; however, under severe stimuli, the excessive accumulation of ROS breaks the cellular homeostasis, resulting in oxidative stress and cellular dysfunctions (Bar-Or et al., 2015; Poprac et al., 2017). Among all the differentiated organs, the gastrointestinal tract is prone to be affected by oxidative stress due to the prolonged luminal oxidant exposure, exogenous stimuli or imbalanced microbiota (Circu and Aw, 2012; Qiao et al., 2013; Diaz-Ochoa et al., 2016). Accumulating evidence has implicated that oxidative stress is associated with various intestinal diseases, such as peptic ulcers, diarrhea, inflammatory bowel disease and colon cancer (Granot and Kohen, 2004; Akaike et al., 2014; Bhattacharyya et al., 2014; Pereira et al., 2015). Diquat [(1,1’-ethylene-2,2’-dipyridylium, DQ)] is a redox cycling bipyridylium herbicide that utilizes molecular oxygen to generate ROS and induces acute oxidative damages (Gallagher et al., 1995). The target organs are liver, kidney and intestine (Rawlings et al., 1994; Lu et al., 2010). Due to its remarkable effect, DQ has been widely used to establish oxidative stress models both *in vivo* and *in vitro* (Smith et al., 1985; Song et al., 2017).

Autophagy, an intracellular catabolic process aiming at engulfing and degrading damaged components, is believed to play a crucial role in response to oxidative stress (Scherz-Shouval and Elazar, 2007). Autophagy machinery contributes to eliminating oxidized macromolecules and damaged organelles such as mitochondria and endoplasmic reticulum (Yorimitsu and Klionsky, 2007; Kurihara et al., 2012; Li et al., 2012). Moreover, autophagy-mediated antioxidant pathways are reported to sense cellular damage in the context of pathogenesis (Lee et al., 2012). The oxidative stress inducers ROS facilitate several stages of autophagy process including initiation and autophagosome elongation (Lee et al., 2012). In turn, the wide-ranging damages caused by ROS can be prevented by autophagy. Mitochondria are the major sites that are responsible for more than 90% of ROS generation (Skulachev, 2012). Studies showed that the special mitochondrial autophagy, termed “mitophagy”, is a key approach for the clearance of ROS (Li et al., 2015). Defects of autophagy may result in abnormal physiological functions and severe oxidative stress. The induction of autophagy requires numerous proteins. Almost 40 autophagy-related (Atg) proteins have been identified to orchestrate this process (Shibutani et al., 2015). The autophagy markers are microtubule-associated protein 1 light chain 3 (LC3, a mammalian homolog of yeast Atg8), Beclin1 (Atg6), and a ubiquitinated protein called P62 (Itakura and Mizushima, 2011). There are several signal pathways regulating autophagy. The most studied pathway is AKT/mTOR, which functions as a key regulator in autophagy induction (Heras-Sandoval et al., 2014). MAPKs, including JNK, p38, and ERK1/2, have also been reported to play an important role in autophagy by modulating the expression of multiple Atg genes (Zhou et al., 2015).

Probiotic strains, such as *Bifidobacterium animalis*, *Lactobacillus rhamnosus*, and *Bacillus* LBP32 have been shown to exhibit potent antioxidant capacity and alleviate oxidative damages both *in vivo* and *in vitro* (Amaretti et al., 2013; Diao et al., 2014; Singh et al., 2017). The possible mechanisms underlying antioxidant effects of probiotics include: (1) self-secretion of antioxidant metabolites, (2) modulation of the antioxidases activities, and (3) down-regulation of enzyme activities that mediate ROS production (Wang et al., 2017b). However, only a few studies have elaborated on how probiotics modulate anti-oxidative status of the host and the involved molecular signaling pathways (Petrof et al., 2004; Gao et al., 2013). In recent years, investigators have found that probiotics can regulate autophagy to protect the host from stresses and pathogen infections. Kim et al. (2010) showed that cell-bound exopolysaccharides from *Lactobacillus acidophilus* induce autophagy to exert an antitumor effect. *L. rhamnosus* modulates the balance between apoptosis and autophagy in zebrafish (Gioacchini et al., 2013). In contrast, *L. rhamnosus GG* suppresses autophagy to protect against *Salmonella infantis* infection in the pig intestine (Zhang et al., 2018). *Bacillus*, a type of probiotic, is widely used to prevent gastrointestinal disorders and improve animal growth performance (Ai et al., 2011; Baker et al., 2013; Zhang and Kim, 2014). Recent studies also showed that some *Bacillus* strains are beneficial in protecting against oxidative stress (Bai et al., 2016; Wang et al., 2017a). However, whether autophagy takes part in *Bacillus*-mediated antioxidation remains unknown. Thus, in this study, our aim is to evaluate the antioxidant capacities of two probiotic strains, *Bacillus amyloliquefaciens* SC06 and *Bacillus licheniformis* SC08 in rats under conditions of DQ-induced oxidative stress, and then use IEC-6 cells to test the hypothesis that autophagy is involved in the mechanisms of *Bacillus*-mediated antioxidation.

## Materials and Methods

### Bacteria Preparation

Probiotics *B. amyloliquefaciens* SC06 and *B. licheniformis* SC08 were stored in our Lab, which were, respectively isolated from soil and a commercial capsule (Ji et al., 2013; Wang et al., 2017a). The identification of these strains were confirmed by 16SRNA sequencing. The *B. amyloliquefaciens* SC06 was also preserved at the China Center for Type Culture Collection (CCTCC, No: M2012280). Both strains were cultured overnight in Luria-Bertani (LB) broth at 37°C and collected by centrifugation. Bacteria were washed and diluted to 10^7^ cfu/mL in 0.9% saline or suspended at 10^8^ cfu/mL in cell culture medium.

### Animal Experimental Design

Sixty 4-week old male SD rats, purchased from Slac Laboratory Animal Co., Ltd. (Shanghai, China), were housed in an air-conditioned room on a 12-h light/dark cycle and fed a normal diet. After 7 days of acclimatization, sixty rats were randomly divided into three groups (*n* = 20) and intragastrically gavaged with 1 mL of 0.9% saline or 10^7^ cfu/mL SC06 or SC08 on a daily basis as previously described (Ichikawa et al., 1999). Rats were pretreated with either *Bacillus* for a 24-day time period, similar to the previous rat studies with probiotics (Peran et al., 2006; McKernan et al., 2010). The body weight and feed intake were monitored every 4 days. After 24-day administration, half of the rats in each group received an intraperitoneal injection of 12 mg/kg BW DQ in 0.9% saline to induce oxidative stress (Liu et al., 2016), and the remaining rats received the same volume of 0.9% saline. 48 h later, the animals were sacrificed by anesthesia. Samples were collected, frozen in liquid nitrogen, and stored at −80°C.

### Establishment of an Oxidative Stress Model in IEC-6 Cells

The rat intestinal epithelial cell line IEC-6 was purchased from American Type Culture Collection (ATCC, Rockville, United States) and cultured in DMEM (HyClone, Logan, United States) supplemented with 10% FBS (Australian origin, Gibco, Grand Island, United States) and 1% antibiotics at 37°C. The DQ-induced oxidative stress model was evaluated by a cell counting kit 8 (CCK-8, Beyotime, Shanghai, China). Briefly, after overnight culture in 96-well plates, IEC-6 cells were treated with DQ at various concentrations (0, 10, 20, 40, 60, 80, 100, and 200 μmol/L) for 12 h (Song et al., 2017). To determine cell viability, 10 μL of CCK-8 assay solution was added to each well, and the plates were incubated for 1 h. Thereafter, OD values of the wells were measured using a SpectraMax M5 (Sunnyvale, United States). The LD_50_ of DQ was calculated by probability unit based on the CCK-8 assay, and the optimal concentration was selected to establish the oxidative stress model. Afterward, IEC-6 cells were pretreated with 10^8^ cfu/mL SC06 as previously described (Wang et al., 2017a), and then exposed to DQ for 12 h. Samples were assayed to examine cell cytotoxicity by CCK-8 or LDH kits.

### Intestinal Morphological Analysis

For optical microscope observations, rat middle jejunal samples were fixed in 4% paraformaldehyde in PBS, embedded in paraffin, sliced and stained with hematoxylin and eosin (H&E). Images were obtained, and the villus length and crypt depth were measured using an Olympus microsystem (Tokyo, Japan). For transmission electron microscopy (TEM) analysis, specimens were first fixed with 2.5% glutaraldehyde in PBS and then postfixed with 1% OsO_4_ buffer, followed by dehydration in a graded series of ethanol. After infiltration, embedding and ultrathin sectioning, samples were observed by a Hitachi Model H-7650 transmission electron microscope (Tokyo, Japan).

### Apoptosis Analysis

#### TUNEL Assay

Rat jejunum and IEC-6 cells were analyzed by terminal deoxynucleotidyl transferase dUTP nick end labeling (TUNEL) assays. The paraffin-embedded jejunal sections were deparaffinized, hydrated, incubated with proteinase K, and treated with terminal deoxynucleotidyl transferase (TDT) and biotinylated nucleotides. Slides were then treated with saline-sodium citrate buffer, 6% hydrogen peroxide, streptavidin-HRP conjugate and DAB substrate solution, and finally counterstained in hematoxylin solution. Images were obtained with an Olympus microsystem. IEC-6 cells were analyzed using a TUNEL BrightGreen Apoptosis Detection Kit (Vazyme, Nanjing, China) according to the manufacturer’s protocol. Briefly, slides were fixed with 4% paraformaldehyde, incubated with 20 μg/mL proteinase K solution, and treated with BrightGreen labeling mix and recombinant TDT enzyme. Finally, the stained cells were immediately visualized using an Olympus fluorescence microscope.

#### FITC Annexin V/Dead Cell Apoptosis Assay

Apoptotic cells were detected by a flow cytometric FITC Annexin V/Dead Cell Apoptosis Kit (Invitrogen, Carlsbad, United States). Cells were harvested immediately after treatment and suspended in 1 × annexin binding buffer. Then, 5 μL of FITC annexin V and 1 μL of PI working solution were added to 100 μL of cell suspension, and the mixture was incubated for 15 min. The stained cells were measured by a FC500 flow cytometer (Beckman Coulter, Fullerton, United States) to distinguish early apoptotic (annexin V+, PI–) and late apoptotic cells (annexin V+, PI+) from viable (annexin V–, PI–) and necrotic cells (annexin V–, PI+).

### Western Blot

Rat jejunal mucosal samples were collected on sterile glass slides and homogenized in RIPA buffer (Beyotime, Shanghai, China) containing 1% PMSF and phosphatase inhibitors (Sigma-Aldrich, St. Louis, United States), while cell samples were directly lysed with RIPA buffer. All samples remained on ice for 30 min and were then centrifuged for 10 min at 12,000 rpm and 4°C. The supernatant was collected, and the protein concentration was determined using a BCA kit (Beyotime, Shanghai, China). After denaturation, proteins from each sample were separated using 8, 12 or 15% SDS-polyacrylamide gels and transferred to polyvinylidene difluoride (PVDF) membranes (Roche, Mannheim, Germany). After being blocked with 5% non-fat milk in TBS at room temperature for 2 h, the membranes were incubated with primary antibody overnight at 4°C. Primary antibodies against Bax, cleaved caspase-3, Beclin1, SQSTM/p62, phospho (p)-mTOR, mTOR, p-AKT, AKT, p-p38, and p-c-jun N-terminal kinase (JNK) were purchased from Cell Signaling Technology (Danvers, United States). Primary antibodies targeting Bcl2, and LC3B were obtained from Abcam (Cambridge, United States). P-(ERK1/2) and anti-ERK1 antibodies were purchased from BD Biosciences (San Jose, United States), while β-actin, SAPK/JNK and p38 antibodies were purchased from Beyotime (Shanghai, China). After the membranes were incubated with secondary antibody for 1 h, the immunoreactive bands were detected with an ECL system (Tanon, Shanghai, China). The relative band density was determined using ImageJ software.

### Detection of Antioxidant Activities

The levels of malondialdehyde (MDA), superoxide dismutase (SOD), total antioxidant capacity (T-AOC), glutathione (GSH)/oxidized glutathione (GSSG), and GSH-Px in rat jejunal mucosa or IEC-6 cell lysates were determined. All assays were carried out according to the manufacturer’s instructions (Jiancheng Bioengineering Institute, Nanjing, China).

### ROS Generation and ΔΨm Analysis

ROS levels in treated IEC-6 cells were detected using a ROS assay kit (Beyotime, Shanghai, China). Briefly, cells were incubated with 10 μM DCFH-DA solution at 37°C for 20 min. Then, cells were washed with FBS-free medium and harvested in 0.04% EDTA solution. The mitochondrial membrane potential (ΔΨm) was detected with a JC-1 probe using a kit (Beyotime, China). All fluorescence signals were monitored using the SpectraMax M5 (Sunnyvale, United States).

### Immunofluorescence Analysis

Cells were inoculated on coverslips in 12-well plates and cultured overnight. After pretreatment with 10^8^ cfu/mL SC06 for 6 h and incubation with DQ for 12 h, cells were fixed with cold methanol for 5 min and blocked with 2.5% BSA at room temperature for 2 h. Then, samples were incubated with anti-LC3B antibody overnight at 4°C. Next, cells were stained with Alexa Fluor 488-conjugated antibody and DAPI solution. Images were obtained with an Olympus laser scanning confocal microscope (Olympus BX61W1-FV1000, Tokyo, Japan).

### Statistical Analysis

All data are expressed as the mean ± standard deviation (SD). Statistical significance was determined by one-way ANOVA or two-tailed Student’s *t*-test using SPSS 20.0 statistical software (SPSS Inc., Chicago, United States). The LD_50_ of DQ to IEC-6 cells was calculated using the probit method in SPSS 20.0. *P* < 0.05 indicated statistical significance. Figures were prepared using Origin 8.0 software (Origin Lab Corporation).

## Results

### Effects of *Bacillus* on the Growth Performance of Rats

**FIGURE 1:**
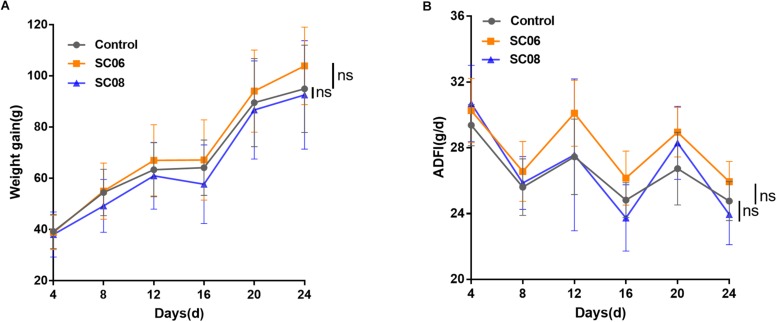
The growth performance of rats administered *Bacillus*. Sixty rats were randomly divided into three groups (*n* = 20 each) and orally administered 1 mL of 0.9% NaCl or 10^7^ cfu/mL *Bacillus* SC06 or SC08 every day. **(A)** The body weight of each rat was measured every 4 days. Weight gain was calculated as (body weight of each rat on day N) - (body weight of each rat on day 0). **(B)** The average daily feed intake (ADFI) was calculated by (total feed intake in 4 days)/20/4. Data of each time point were analyzed using two-tailed Student’s *t*-test (*n* = 20). ns, no significance.

As shown in Figure 1A, compared with the control group, rats administrated *Bacillus* SC06 experienced an increased trend in the weight gain from the 8th day, while SC08 group exhibited a decreased trend from the 4th day; however, the differences were not significant (*p* > 0.05, respectively). The average daily feed intake (ADFI) fluctuated among all the groups, and no significance was shown in *Bacillus*- treated rats (*p* > 0.05) (Figure 1B). These results indicated that supplementation with *Bacillus* SC06 or SC08 did not elicit significant changes on the growth performance of rats.

### *Bacillus* SC06 Attenuated Intestinal Mucosal Injuries of Rats Under Oxidative Stress

**FIGURE 2:**
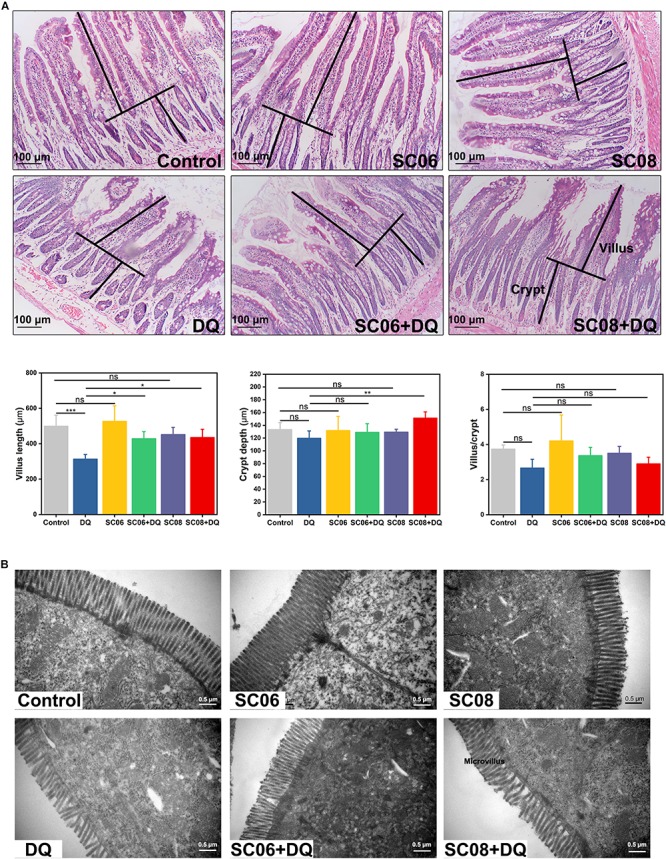
Histomorphometric analysis of the rat jejunum after DQ treatment. **(A)** Hematoxylin and eosin (H&E) staining, 100 × magnification, scale bar: 100 μm. The villus length and crypt depth were measured as shown in the image. The villus length, crypt depth, and villus/crypt ratio were analyzed from the top images. Data were analyzed using one-way ANOVA Tukey’s test (*n* = 10), ^∗^*p* < 0.05, ^∗∗^*p* < 0.01 and ^∗∗∗^*p* < 0.001; ns, no significance (*p* > 0.05). **(B)** Transmission electron microscopy (TEM) analysis of jejunum microvilli. Images were observed at 30,000 × magnification, scale bar: 0.5 μm.

The gastrointestinal tract of rats showed apparent dilation and severe congestion after DQ exposure (Supplementary Figure S1). Histological examination of jejunum showed that the intestinal villi were seriously damaged in response to DQ treatment (Figure 2A), as indicated by shortened intestinal villi and erosion of the mucosal layer. However, administration of probiotics, particularly SC06, attenuated the degree of tissue injury. Statistical results showed that DQ exposure significantly decreased the villus length (*p* < 0.001), while *Bacillus* pretreatments could markedly reverse this trend (*p* < 0.05). The crypt depth only increased in SC08 + DQ group (*p* < 0.01), while no significant differences were observed in the villus/crypt ratio (*p* > 0.05). Similar results were also shown in the ultrastructure of epithelial cells visualized by TEM. As displayed in Figure 2B, DQ exposure significantly reduced microvillus height and impaired jejunal brush border. Additionally, the microvilli were sparse and irregular in DQ group, whereas they were relatively longer and more regular in SC06 + DQ group. These results suggested that *Bacillus*, particularly SC06 could effectively improve rat intestinal morphology and integrity under conditions of DQ-induced oxidative damages.

### *Bacillus* SC06 Decreased Oxidative Stress-Induced Apoptosis of Rat Intestine

**FIGURE 3:**
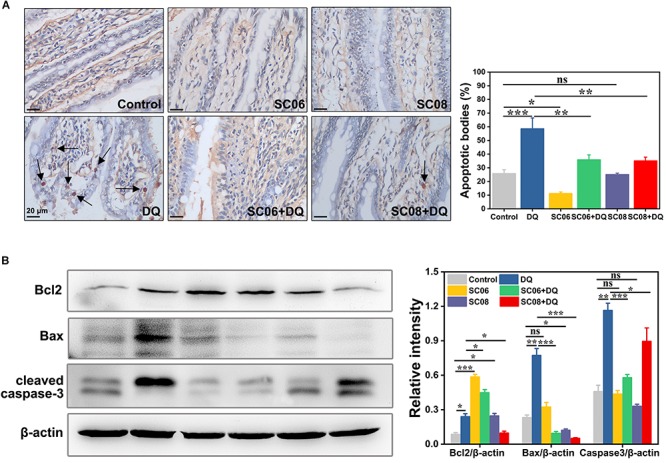
*Bacillus* protected rats from oxidative stress-induced apoptosis. **(A)** Jejunal sections from each group were stained with a terminal deoxynucleotide transferase dUTP nick end labeling (TUNEL) kit. Pictures were captured at 400 × magnification, scale bar: 20 μm. The black arrow marks apoptotic bodies (brown puncta). The number of apoptotic bodies were statistically analyzed by Image J. **(B)** Protein lysates from jejunal mucosa were used to detect the expression of Bcl2, Bax, cleaved caspase-3 by western blot. The ratios of Bcl2, Bax and cleaved caspase-3 to β-actin were analyzed using ImageJ. Data are presented as the mean ± SD (*n* = 3) using one-way ANOVA with Tukey’s test, ^∗^*p* < 0.05, ^∗∗^*p* < 0.01, and ^∗∗∗^*p* < 0.001; ns, no significance (*p* > 0.05).

Oxidative stress is believed to easily cause apoptotic cell death. TUNEL assay was conducted to identify the characteristic of the early stage of apoptosis in rat jejunum. Results indicated that there were few apoptotic bodies (brown granules) in the control, SC06 and SC08 groups, whereas numerous positive cells were observed in DQ- treated group (*p* < 0.001) (Figure 3A). When rats were pretreated with SC06 or SC08, the number of TUNEL-positive cells appeared to decline significantly (*p* < 0.01). The probiotic-exerted anti-apoptosis was further comfirmed by detecting the expression of apoptotic-related proteins. Bcl2 is a crucial repressor of apoptosis, while Bax and cleaved caspase-3 are the promotors (Del Poeta et al., 2003). As shown in Figure 3B, SC06 administration significantly upregulated Bcl2 expression (*p* < 0.001), which even remained high levels after DQ injection (*p* < 0.001). In SC08 group, the expression of Bcl2 increased slightly but decreased dramatically after DQ treatment (*p* < 0.05). The expression of Bax was markedly elevated in the DQ group but was decreased by pretreatment with both probiotics (*p* < 0.001). DQ exposure significantly up-regulated the level of cleaved caspase-3 (*p* < 0.001), whereas SC06 pretreatment markedly reduced it (*p* < 0.001). These findings suggested that probiotics, particularly SC06 could significantly decrease oxidative stress- induced apoptosis.

### *Bacillus* SC06 Exerted Better Antioxidant Properties in Rat Jejunum

**FIGURE 4:**
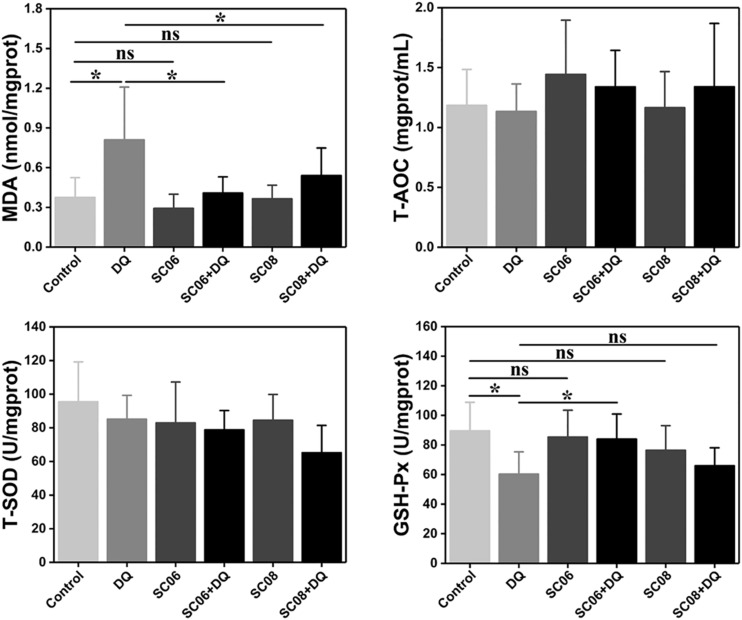
*Bacillus* enhanced antioxidant properties in the rat jejunum. The antioxidant levels were determined by measuring MDA, T-AOC, T-SOD and GSH-Px. Data are presented as the mean ± SD (*n* = 8), one-way ANOVA with Tukey’s test, ^∗^*p* < 0.05; ns, no significance (*p* > 0.05).

Free radicals can cause membrane lipid peroxidation and MDA is the representative biomarker. As shown in Figure 4, the MDA concentration in DQ group was significantly increased (0.81 ± 0.43 nmol/mgprotein, *p* < 0.05), while the values were markedly dropped by pretreatment with either probiotic (0.41 ± 0.13 and 0.54 ± 0.22 nmol/mgprotein, respectively, *p* < 0.05). Unexpectedly, T-AOC and T-SOD activities showed no obvious changes in any of the groups (*p* > 0.05). GSH-Px is one of the major antioxidant enzymes that protect against oxidative stress. In the present study, DQ exposure down-regulated GSH-Px activity, with the value of 60.30 ± 6.08 U/mgprotein (*p* < 0.05). However, this trend was significantly altered by SC06 pretreatment (83.91 ± 1.82 U/mgprotein, *p* < 0.05), but not by SC08 (65.88 ± 1.30 U/mgprotein, *p* > 0.05). These results suggested that probiotic SC06 showed better antioxidant capacity than SC08 under DQ exposure.

### The Establishment of DQ-Induced Oxidative Stress Model in IEC-6 Cells

**FIGURE 5:**
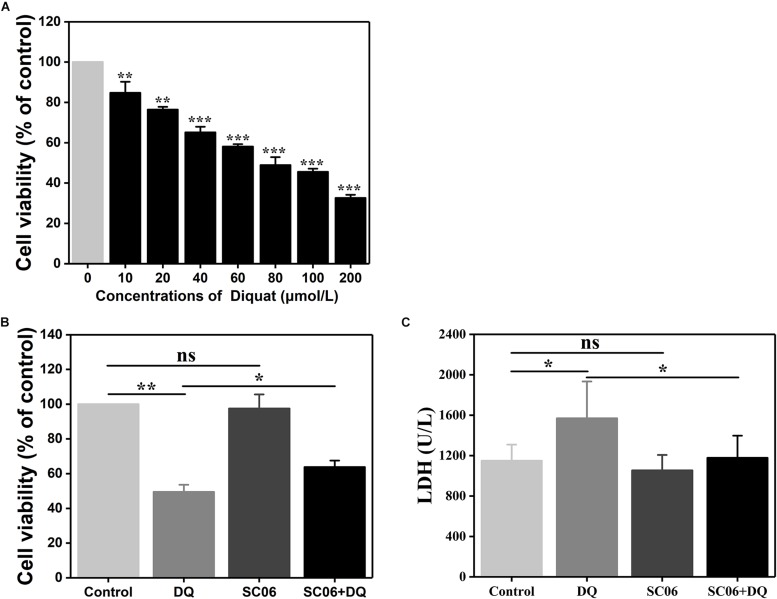
The establishment of DQ-induced oxidative stress model in IEC-6 cells. **(A)** IEC-6 cells were treated with different doses of DQ (0, 10, 20, 40, 60, 80, 100, or 200 μmol/L) for 12 h. Cell viability was evaluated by CCK-8 test. Data are presented as mean ± SD (*n* = 9) using two-tailed Student’s *t*-test, ^∗∗^*p* < 0.01 and ^∗∗∗^*p* < 0.001. **(B,C)** Cells were preincubated with 10^8^ cfu/mL SC06 for 6 h and then exposed to 80 μmol/L DQ for 12 h. LDH release in the supernatant was assayed using a kit. Data are presented of three individual experiments with similar results using one-way ANOVA with Tukey’s test, ^∗^*p* < 0.05 and ^∗∗^*p* < 0.01; ns, no significance (*p* > 0.05).

We established a DQ-induced oxidative stress model in IEC-6 cells to further investigate SC06-exerted antioxidant functions and the involved molecular mechanisms. As shown in Figure 5A, DQ decreased IEC-6 cell viability in a dose-dependent manner. Compared to the control, cells treated with 60 or 80 μmol/L DQ showed significant reduced viability by 58.09 ± 1.28% and 48.91 ± 4.24%, respectively (*p* < 0.001). The LD_50_ for DQ in IEC-6 cells was calculated to be 82.56 μmol/L using the probit method. Therefore, 80 μmol/L DQ was used in the following experiment. Subsequently, we examined the protective effects of probiotic SC06 on cell viability and LDH release. Statistical analysis revealed that DQ exposure markedly decreased cell viability by 49.50 ± 4.29% (*p* < 0.01), while pretreated with SC06 significantly reversed this reduction to 63.82 ± 3.93% (*p* < 0.05) (Figure 5B). LDH leakage is a biomarker of cell damage. Similarly, DQ treatment significantly increased LDH release, but this trend was inhibited by SC06 pretreatment (*p* < 0.05) (Figure 5C).

### *Bacillus* SC06 Significantly Increased Antioxidant Levels and Modulated ROS Production and ΔΨm in IEC-6 Cells

**FIGURE 6:**
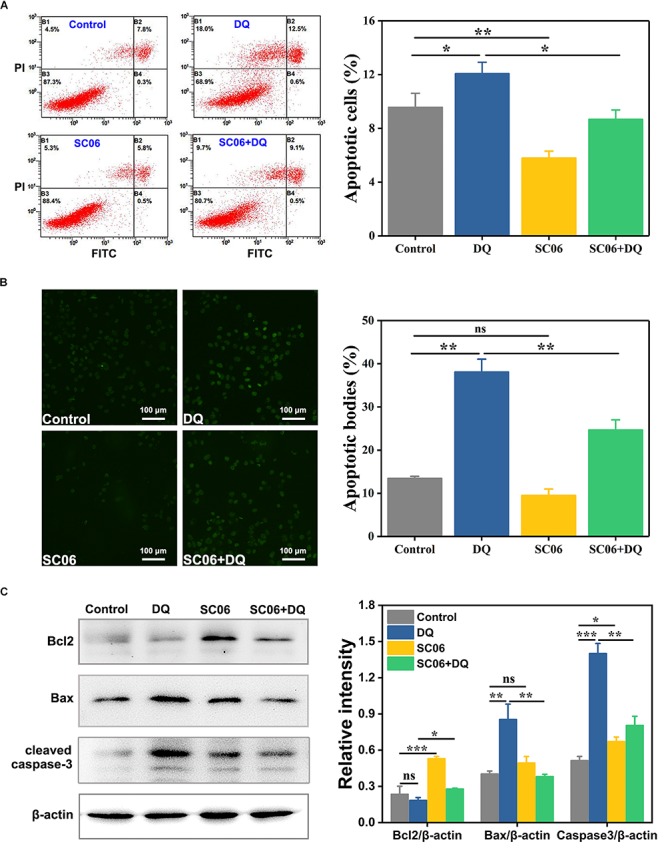
*Bacillus* SC06 inhibited apoptosis in IEC-6 cells under DQ exposure. **(A)** FITC Annexin V apoptosis assay. Cells were pretreated with 10^8^ cfu/mL SC06 for 6 h and then exposed to 80 μmol/L DQ for 12 h. Apoptotic rates were determined using a FITC Annexin V/PI apoptosis kit and analyzed by flow cytometry. **(B)** TUNEL assay. After treatment with 10^8^ cfu/mL SC06 and 80 μmol/L DQ, cells were stained using a BrightGreen apoptosis detection kit. Pictures were observed *at 200 × magnification. Scale bar: 100 μm. The number of apoptotic bodies were statistically analyzed by Image J. **(C)** The protein expression of Bcl2, Bax and cleaved caspase-3 in cell lysates was examined by western blot. The ratios of Bcl2, Bax and cleaved caspase-3 to β-actin were analyzed using ImageJ. Data are presented as the mean ± SD (*n* = 3) using one-way ANOVA with Tukey’s test, ^∗^*p* < 0.05, ^∗∗^*p* < 0.01, and ^∗∗∗^*p* < 0.001; ns, no significance (*p* > 0.05).*

To investigate the effect of *Bacillus* SC06 on anti-apoptosis in IEC-6 cells, we first conducted the FITC annexin V/apoptosis assay. As shown in Figure 6A, pretreatment with SC06 markedly decreased DQ-triggered apoptosis (*p* < 0.05). Specifically, the percentages of apoptotic cells (including early and late) in control, DQ, SC06 and SC06 + DQ groups were 9.6 ± 1.3%, 12.1 ± 1.1%, 5.8 ± 0.6%, and 8.7 ± 0.9%, respectively. In addition, the number of necrotic cells was the highest in DQ group (15.3 ± 2.4%). Results from the TUNEL assay showed that compared with control group, DQ treatment dramatically increased the number of apoptotic cells (green puncta) (*p* < 0.01). In contrast, a significant decrease was observed in cells pretreated with SC06 (*p* < 0.01) (Figure 6B). Furthermore, as shown in Figure 6C, SC06 pretreatment markedly upregulated the protein level of Bcl2, compared to DQ group (*p* < 0.05). The expression of Bax were robustly increased after DQ treatment, but this trend was significantly altered by SC06 (*p* < 0.01). DQ exposure dramatically increased the level of cleaved caspase-3 (*p* < 0.001), while SC06 pretreatment markedly down-regulated it (*p* < 0.01). These results indicated that SC06 pretreatment alleviated oxidative stress- induced cell apoptosis in IEC-6 cells.

**FIGURE 7:**
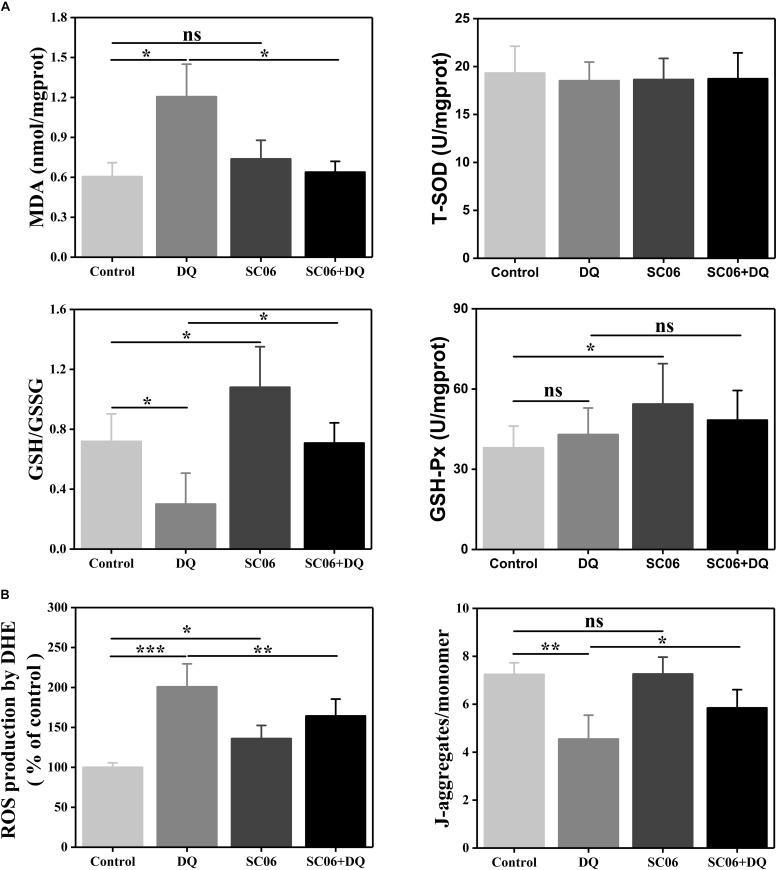
*Bacillus* SC06 increased antioxidant levels in IEC-6 cells. **(A)** Cells were co-cultured with 10^8^ cfu/mL SC06 for 6 h and then treated with 80 μmol/L DQ for 12 h. Antioxidant activity in cell lysates was determined by measuring MDA, GSH/GSSG, T-SOD and GSH-Px. **(B)** ROS levels were measured by DCFH-DA fluorescence assay. Mitochondrial membrane potential (ΔΨm) was detected using JC-1 staining and data are presented as the ratio of red fluorescence (JC-1 aggregates) to green fluorescence (JC-1 monomers). All data are representative of three individual experiments with similar results using one-way ANOVA with Tukey’s test, ^∗^*p* < 0.05, ^∗∗^*p* < 0.01, and ^∗∗∗^*p* < 0.01; ns, no significance (*p* > 0.05).

To further determine the effects of SC06 on alleviating oxidative stress, we examined the levels of antioxidant indices. As shown in Figure 7A, MDA content was significantly increased in DQ group (1.21 ± 0.26 nmol/mgprotein, *p* < 0.05), but markedly reduced in SC06 + DQ group (0.64 ± 0.09 nmol/mgprotein, *p* < 0.05). GSH/GSSG ratio is considered as a powerful biomarker of oxidative stress (Giustarini et al., 2016). In the present study, the GSH/GSSG ratio in DQ-treated cells was dramatically decreased to 0.30 ± 0.22 (*p* < 0.05), while significantly upregulated in SC06-treated cells (1.08 ± 0.29, *p* < 0.05). SC06 pretreatment could markedly inhibit the reduction of GSH/GSSG ratio (0.71 ± 0.14, *p* < 0.05). Similar to the results in rat jejunum, no significant difference in the T-SOD level was observed in any groups of treated cells, while the GSH-Px activity was increased in SC06-treated cells (*p* < 0.05).

Next, we examined ROS production in IEC-6 cells using DCFH-DA fluorescence assay. As shown in Figure 7B, compared with control cells, DQ exposure significantly increased ROS generation (200.83 ± 23.47%, *p* < 0.001), while pretreatment with SC06 markedly inhibited it by 164.36 ± 22.41% (*p* < 0.01). Since ΔΨm loss is a key event in early apoptosis through the mitochondrial apoptotic pathway, we detected the changes in ΔΨm by JC-1 staining. After DQ exposure, the ratio of JC-1 aggregates and monomers was significantly decreased by 4.55 ± 1.05 (*p* < 0.01), but was considerably recovered when pretreated with SC06 (5.85 ± 0.81, *p* < 0.05) (Figure 7B). All the above results illustrated that SC06 pretreatment reduced oxidative damages of IEC-6 cell under DQ exposure.

### *Bacillus* SC06 Attenuated Oxidative Stress by Promoting Autophagy

**FIGURE 8:**
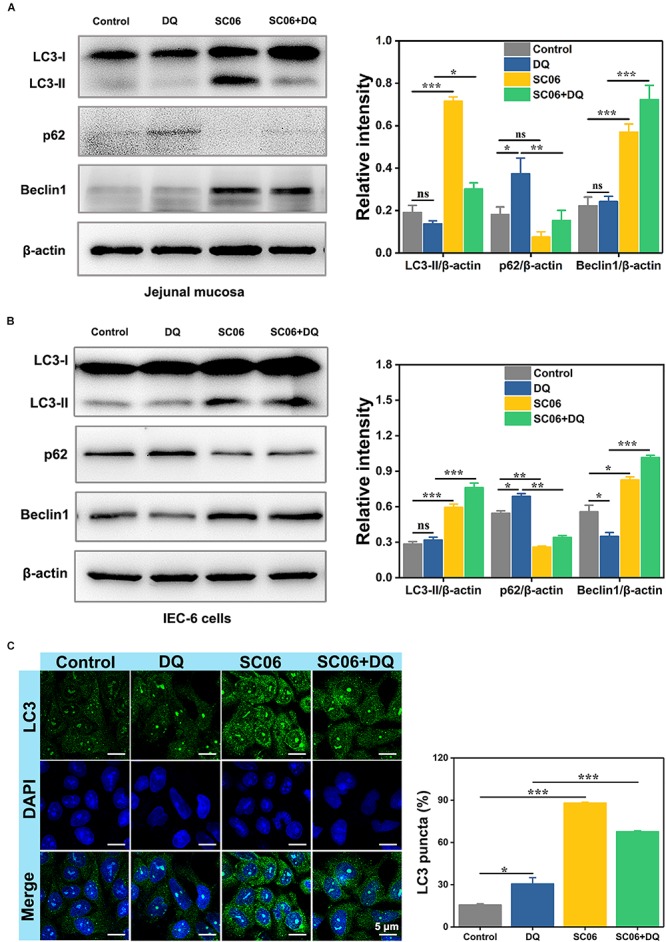
*Bacillus* SC06 alleviated oxidative stress by promoting autophagy. **(A,B)** The protein expression of the autophagy markers LC3, p62 and Beclin1 in rat jejunal mucosa and IEC-6 cells was detected by western blot. The ratios of LC3-II, p62 or Beclin1 to β-actin were analyzed by ImageJ. **(C)** After treatment with 10^8^ cfu/mL SC06 and 80 μmol/L DQ, cells were stained for immunofluorescence analysis and visualized by confocal microscopy. Scale bar: 5 μm. The number of LC3-positive cells (> 3 green puncta) was statistically analyzed. Data are presented as the mean ± SD (*n* = 3) using one-way ANOVA with Tukey’s test, ^∗^*p* < 0.05, ^∗∗^*p* < 0.01, and ^∗∗∗^*p* < 0.001; ns, no significance (*p* > 0.05).

It was reported that autophagy plays a pivotal role in eliminating ROS and inhibiting apoptosis in response to oxidative stress (Bhogal et al., 2012). To explore whether SC06 pretreatment triggered autophagy, we examined the protein levels of autophagic markers LC3-II, p62 and Beclin1 of rat jejunum and IEC-6 cells. As shown in Figure 8A, LC3-II expression dramatically increased in SC06-treated rats (*p* < 0.001). No difference of LC3-II expression was found in DQ group (*p* > 0.05), while there was a marked increase in SC06 + DQ group (*p* < 0.05). DQ treatment significantly inhibited p62 degradation, but this trend was blocked by SC06 administration (*p* < 0.01). Furthermore, SC06 pretreatment markedly upregulated the expression of Beclin1 (*p* < 0.001), compared to DQ group (*p* < 0.001). Consistently, similar results were found in IEC-6 cells (Figure 8B). SC06-induced autophagy was also evaluated based on the percentage of LC3-positive cells using confocal laser scanning microscopy (Figure 8C). Compared with the control group, DQ exposure slightly up-regulated LC3-positive cells (30.57 ± 1.33%, *p* < 0.05). However, when IEC-6 cells were pretreated with SC06, a significant increase was shown in LC3 puncta (67.59 ± 0.90%, *p* < 0.001). Taken together, these findings indicated that SC06 pretreatment could induce autophagy both *in vivo* and *in vitro*.

**FIGURE 9:**
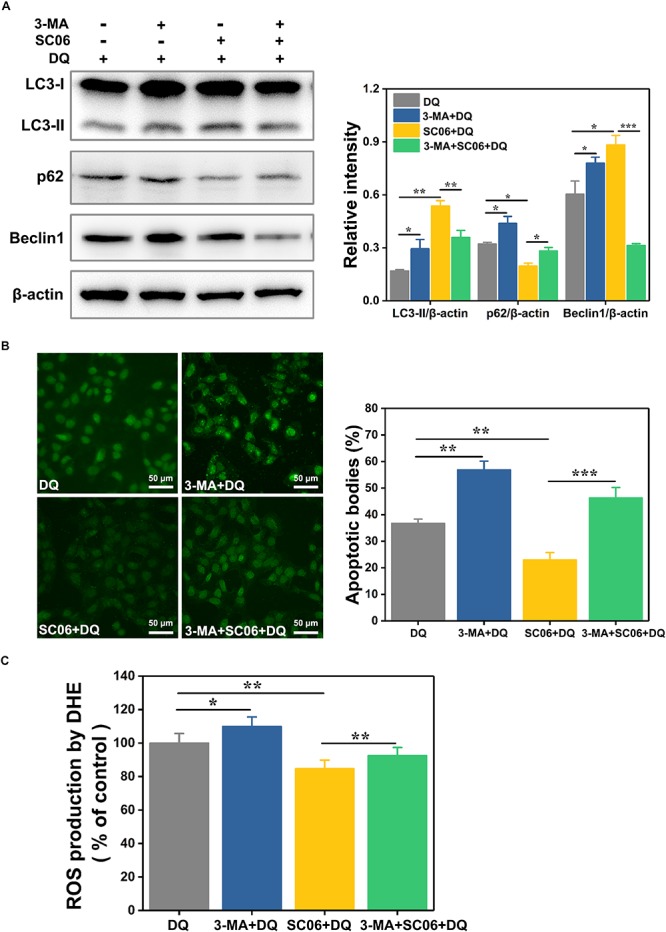
Determination of the relationship between SC06-induced autophagy and antioxidation in IEC-6 cells. **(A)** Cells were pretreated with 3-MA (10 mmol/L, 1 h) and then incubated with 10^8^ cfu/mL SC06 for 6 h. After that, 80 μmol/L DQ was added to all the groups and treated for 12 h. The levels of LC3, p62 and Beclin1 were identified by western blot. The ratios of LC3-II, p62 or Beclin1 to β-actin were calculated using ImageJ. **(B)** After treatment with 3-MA, SC06 and DQ, cells were harvested, stained with the TUNEL kit and observed at 400 × magnification. Scale bar: 50 μm. The apoptotic cells were quantitatively analyzed by Image J. **(C)** The treated cells were collected and incubated with ROS detection solution. The ROS concentration was detected by a fluorescence spectrophotometer. Data are presented as the mean ± SD (*n* = 3) using one-way ANOVA with Tukey’s test, ^∗^*p* < 0.05, ^∗∗^*p* < 0.01, and ^∗∗∗^*p* < 0.001.

To clarify the relationship between autophagy and SC06-mediated protective effects, IEC-6 cells were pretreated with 3-methyladenine (3-MA), a widely used autophagy inhibitor that blocks the class-III PI3K signaling pathway (Wu et al., 2010). As shown in Figure 9A, 3-MA blocked the increased expression of LC3-II and Beclin1 and the degradation of p62 which were induced by SC06 pretreatment, confirming the suppression of autophagy. Thereafter, we examined whether autophagy inhibition affected the capacities of anti-apoptosis and antioxidation of SC06. TUNEL assay showed that SC06 markedly reduced the number of green apoptotic bodies (22.96 ± 3.42%, *p* < 0.01), but this trend was significantly inhibted by 3-MA pretreatment (46.32 ± 4.78, *p* < 0.001) (Figure 9B). Furthermore, ROS production was significantly decreased in SC06 group (84.59 ± 2.76%, *p* < 0.01), but accumulated in 3-MA + SC06 + DQ group (92.52 ± 2.32%, *p* < 0.01) (Figure 9C). These findings confirmed that SC06 triggered autophagy to exert antioxidation and anti-apoptosis effects.

### SC06-Mediated Autophagy Was Triggered by p38 MAPK, Not by AKT/mTOR Signaling Pathway

**FIGURE 10:**
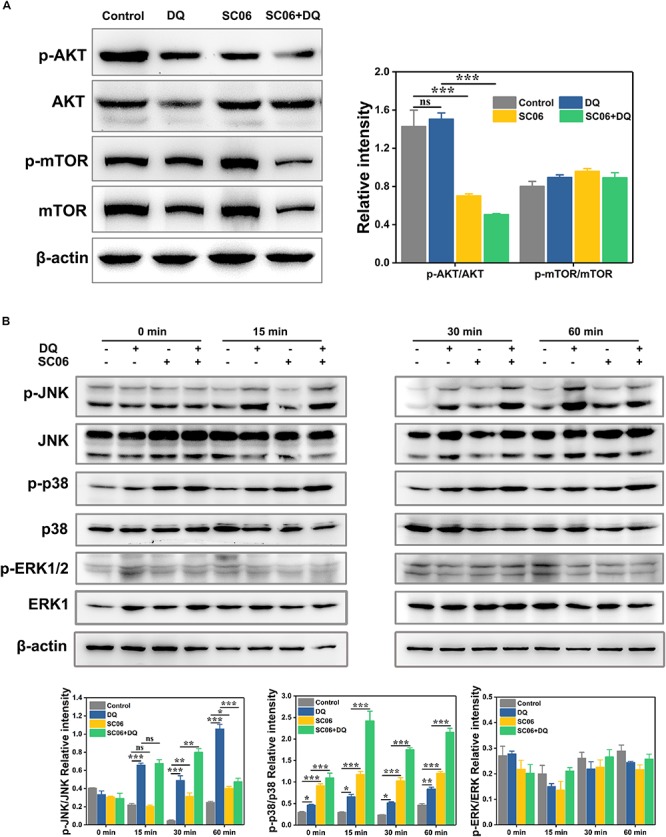
SC06-mediated autophagy was triggered by p38 MAPK, but not by AKT-mTOR signaling pathway in IEC-6 cells. **(A)** Cells were cultured with 10^8^ cfu/mL SC06 and exposed to 80 μmol/L DQ. Then, cells were harvested to detect the expression of p-AKT, anti-AKT, anti-p-mTOR and anti-mTOR. **(B)** Cells were pretreated with SC06 (10^8^ cfu/mL, 6 h) and then exposed to 80 μmol/L DQ for 0, 15, 30, or 60 min. The expression levels of p-JNK, JNK, p-p38, p38, p-ERK and ERK were identified by western blot. The relative intensity of all the protein bands were analyzed using ImageJ. Data are presented as the mean ± SD (*n* = 3) using one-way ANOVA with Tukey’s test, ^∗^*p* < 0.05, ^∗∗^*p* < 0.01, and ^∗∗∗^*p* < 0.001; ns, no significance (*p* > 0.05).

We then investigated the signaling pathways involved in SC06-mediated autophagy. As shown in Figure 10A, compared with the control group, the SC06-treated cells exhibited a significant reduction in p-AKT/AKT levels (*p* < 0.001). However, no significant changes of p-mTOR/mTOR were observed in all the treated groups (*p* > 0.05). These results implied that the AKT/mTOR signaling pathway was independent. We then determined the role of MAPK signaling pathways in SC06-triggered autophagy. As illustrated in Figure 10B, DQ dramatically increased JNK phosphorylation from 15 to 60 min (*p* < 0.001), while a marked decline was shown at 60 min in SC06 + DQ group (*p* < 0.001). A significant accumulation of p-p38 was observed at 0 min and maintained high levels persistently up to 60 min with SC06 pretreatment (*p* < 0.001). Although DQ exposure also upregulated p38 phosphorylation (*p* < 0.05), the levels in SC06 + DQ group were much higher (*p* < 0.001). Neither SC06 nor DQ had a significant effect on p-ERK1/2 MAPK levels (*p* > 0.05).

**FIGURE 11:**
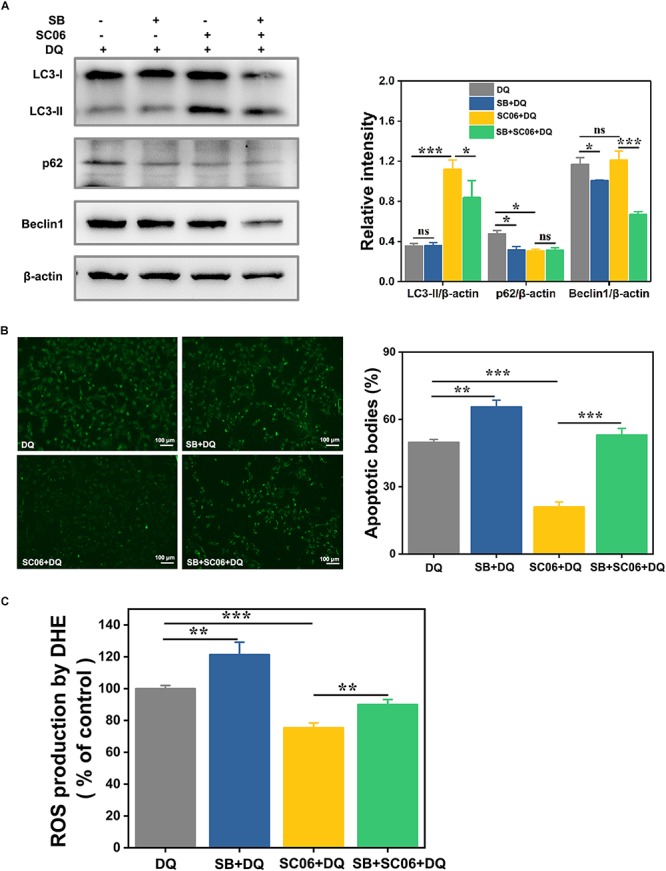
P38 MAPK signaling pathway was involved in SC06-mediated autophagy and antioxidation in IEC-6 cells. Cells were pretreated with 20 μmol/L SB203580 (SB, p38 inhibitor) for 1 h and then cultured with 10^8^ cfu/mL SC06. Thereafter, 80 μmol/L DQ was added to all the cells for another 12 h. **(A)** Cells were harvested to examine the expression of LC3, p62 and Beclin1. The ratios of LC3-II, p62 or Beclin1 to β-actin were calculated using ImageJ. **(B)** Cells were stained with the TUNEL kit and observed at 200 × magnification. Scale bar: 100 μm. The apoptotic bodies were quantitatively analyzed by Image J. **(C)** The treated cells were incubated with ROS detection solution. ROS concentration was detected by a fluorescence spectrophotometer. Data are presented as the mean ± SD (*n* = 3) using one-way ANOVA with Tukey’s test. ^∗^*p* < 0.05, ^∗∗^*p* < 0.01, and ^∗∗∗^*p* < 0.001; ns, no significance (*p* > 0.05).

Next we investigated whether p38 activation participated in SC06-induced autophagy. Cells were preincubated with p38 inhibitor SB203580 (SB) for 1 h before SC06 and DQ treatments. As shown in Figure 11A, compared to DQ group, no changes of LC3-II expression was found in SB + DQ group (*p* > 0.05), while a significant upregulation was shown by SC06 treatment (*p* < 0.001). When compared with SC06 + DQ group, there was a marked reduction of LC3-II level in SB + SC06 + DQ group (*p* < 0.05). SB pretreatment seemingly did not block p62 degradation and even showed a downward trend of p62 expression, while it could dramatically reduce the expression of Beclin1 (*p* < 0.001). Furthermore, we found that SC06 treatment significantly decreased the apoptotic cells but this trend was blocked by SB pretreatment (*p* < 0.001) (Figure 11B). Similarly, SC06-mediated ROS reduction was inhibited when cells were treated with SB (*p* < 0.01) (Figure 11C). These results indicated that SC06 triggered autophagy and antioxidation by activating p38 signaling pathway.

## Discussion

*Bacillus* species have been used for more than 50 years with an OTC medicinal supplement known as Enterogermina^®^ registered 1958 in Italy, while the increasing scientific interests in *Bacillus* as probiotic occurred from the last two decades. *Bacillus* strains are able to produce a variety of enzymes (such as protease, amylase, and lipase), antimicrobial metabolites and bio-peptides (Cutting, 2011). These metabolites contribute to improving nutrient digestion and absorption, inhibiting pathogenic bacteria growth and regulating intestinal microbiota. Furthermore, *Bacillus* spores are capable of survival low gastric pH and completely reaching the intestine, which is not the case for all *Lactobacillus* species (Barbosa et al., 2005; Tuohy et al., 2007). These advantages make *Bacillus* extensively used in humans as dietary supplements and therapeutic medicines, in animals as growth promoters and competitive exclusion agents, and in aquaculture for enhancing shrimp growth and disease-resistance (Cutting, 2011). In the present study, we found that *Bacillus* SC06 was superior to SC08 in alleviating oxidative damages, as evidenced by the improved intestinal epithelial structure and the increased antioxidant and anti-apoptotic properties. Our results further confirmed the concept that the health benefits of probiotics should be concerned at the level of genus, species and strains, because one probiotic is not likely to possess all the proposed effectiveness and the potential application of probiotics is determined by its ability to present their most characteristic features (Bermudez-Brito et al., 2012; Kumar Bajaj et al., 2015). Consistent with our results, in one study it was reported that only three probiotics enhanced antioxidant defenses in the host among thirty-four strains of lactic acid bacteria, indicating the antioxidative properties are strain specific (Amaretti et al., 2013).

Diquat exerts oxidative stress via free radical chain reactions. During redox cycling, DQ is easily converted to radical cations. In the presence of molecular oxygen (O2), the radicals rapidly form superoxide anion radical (O_2_⋅^–^) and then generate hydrogen peroxide (H_2_O_2_) and hydroxyl radicals (HO⋅) (Fussell et al., 2011). These ROS induce lipid peroxidation and potentially cause cell apoptosis (Jones and Vale, 2000). MDA is the indicator of lipid peroxidation. We found that DQ exposure significantly increased MDA levels in rat intestine and IEC-6 cells, while *Bacillus* pretreatment markedly reversed this up-regulation, suggesting the protective role of *Bacillus* in alleviating DQ-induced oxidative damages. Interestingly, our results showed that both DQ and *Bacillus* could not improve T-AOC and increase SOD levels. SOD is a major antioxidant enzyme that eliminates ROS and terminates lipid peroxidation; however, during acute injuries, SOD activity might be defected, leading to the aggragated lipid peroxidation, DNA damage and cell dysfunction (McCord and Edeas, 2005). Consistent with our result, one study found that the SOD levels of rats showed no significant changes after 30 min, 24 h, and 7 days under DQ exposure (Djukic et al., 2012). However, another study reported that DQ caused a dramatic decrease of SOD in plasma of rats (Lu et al., 2010). Thus, we speculate that the SOD activity depends on the status and severity of oxidative stress. This might also be the possible reasons for the different results of GSH-Px levels we detected in rat intestine and IEC-6 cells during DQ treatment. Furthermore, we found that *Bacillus* SC06 pretreatment increased GSH-Px activity, indicating its potential in improving antioxidant levels, but the underlying mechanism needs further studied. It was reported that DQ-induced oxidative stress possibly due to the depletion of GSH (Smith et al., 1985). By reacting with ROS, GSH acts as an electron donor and is converted to its oxidized form GSSG. Thus, the GSH/GSSG ratio has been used to indicate oxidative stress (Cao et al., 2013). In our study, *Bacillus* pretreatment increased GSSG/GSH, suggesting the attenuation of the severity of oxidative stress.

Mitochondrion is the most common source of ROS generation (Curtin et al., 2002). Under normal conditions, only a small percentage of electrons escape the electron transport chain (ETC) and react with O_2_ to form superoxide (O_2_^–^), while under stressed circumstances, antioxidants become overwhelmed or exhausted, resulting in uncontrolled ROS production (Sanderson et al., 2013). An increased ROS production causes mitochondrial damage and finally leads to apoptotic cell death (Leon et al., 2005; Ott et al., 2007). The ΔΨm represents the overall charge across the inner mitochondrial membrane and controls ROS production, while the induction of apoptosis involves the transient hyperpolarization of ΔΨm (Kadenbach et al., 2004). In our study, we found that *Bacillus* SC06 significantly decreased DQ-mediated ROS production and altered the trend of ΔΨm loss. These results indicated that *Bacillus* SC06 could effectively improve mitochondrial functions. There are two major apoptotic pathways, and one is the intrinsic mitochondrial pathway that is regulated by the Bcl2 protein family (Wang et al., 2012). The Bcl2 protein family consists of pro-apoptotic (Bax and Bak1) and anti-apoptotic proteins (e.g., Bcl2, Bcl-xL, and Mcl-1) (Merry and Korsmeyer, 1997). In the current study, DQ exposure significantly increased Bax expression both *in vivo* and *in vitro.* DQ induces oxidative stress by producing ROS (Gallagher et al., 1995), while ROS generation can indirectly induce Bax expression and finally cause apoptotic cell death (Cheng et al., 2009). When rats or IEC-6 cells were pretreated with *Bacillus*, the level of Bax dramatically decreased, which further indicated the inhibition of ROS production by *Bacillus*. Bcl-2 blocks apoptosis pathway by interacting with Bax and inhibiting mitochondria permeabilization (O’Neill et al., 2016). We found that SC06 pretreatment markedly increased Bcl2 expression, suggesting its strong anti-apoptotic capacity. Noticeably, DQ treatment also slightly increased Bcl2 expression. This result was consistent with a recent report describing that doxorubicin exposure, which also induced oxidative stress, elicited significantly higher up-regulation of Bcl2 expression in Calu-1 cells (Farhane et al., 2017). The underlying reason might be that upon oxidative stress, cells can spontaneously activate protein activities and initiate the cellular anti-apoptotic resistance mechanism to protect oxidative stress. Caspase-3 activation is the executor of apoptosis. The increased level of cleaved caspase-3 reveals the imbalance between pro-apoptotic and anti-apoptotic proteins (Green and Reed, 1998). In our study, SC06 significantly down-regulated the increased expression of cleaved caspase-3 expression caused by DQ exposure, indicating the inhibition of apoptosis. All the above findings showed that *Bacillus* SC06 pretreatment decreased oxidative stress induced-apoptosis via reducing ROS production, mitochondrial dysfunctions and the expression of apoptotic proteins.

Autophagy serves as a defense mechanism to clear oxidatively damaged proteins and organelles during oxidative stress (Scherz-Shouval and Elazar, 2007). As a prosurvival mechanism, autophagy can block or delay apoptotic cell death. Results in our study showed that *Bacillus* SC06 triggered autophagy-related processes, including up-regulating LC3 and Beclin1, degrading p62 and increasing LC3 puncta. Althougt a slight increase of LC3- positive cells was found in DQ group, DQ exposure could not activate the expression of autophagic proteins, revealing its defect in autophagy induction. When using 3-MA to block autophagy, the number of apoptotic cells and ROS production significantly increased. These findings revealed that *Bacillus* SC06-induced autophagy contributes to decreasing oxidative stress and apoptosis. In agreement with our results, many other antioxidants have been showed to protect against oxidative stress and apoptosis by inducing autophagy. For instance, cobalt protoporphyrin accelerates autophagy and suppresses LPS-induced oxidative damage in the rat liver (Unuma et al., 2013). Curcumin protects cells against oxidative stress by inhibiting apoptosis and inducing autophagy (Guo et al., 2016). To our knowledge, this is the first study to show that *Bacillus* can directly activate host autophagy to alleviate oxidative stress and apoptosis. Differently, previous investigations showed that *Bacillus* improved host antioxidant ability in an indirect way by stimulating the secretion of antioxidant enzymes and antioxidants that removed excess free radicals and regulate the body’s free radial balance (Li et al., 2012).

AKT/mTOR pathway was first explored to elucidate the underlying signaling pathways participating in the regulation of autophagy by *Bacillus* SC06. Inhibition of AKT/mTOR pathway is regarded as the classic event in the modulation of autophagic process (Heras-Sandoval et al., 2014). In the present study, although AKT phosphorylation was decreased by SC06 treatment, there was no difference in p-mTOR levels, indicating that the AKT/mTOR pathway was not involved in SC06-induced autophagy. AKT is a central player in signal transduction pathways, and accumulating studies have shown that AKT activation can increase oxidative stress and apoptosis (Byon et al., 2008; Martelli et al., 2012). AKT increases intracellular ROS levels by stimulating oxidative metabolism in mitochondria and by repressing antioxidant enzyme expression via FoxO phosphorylation (Nogueira et al., 2008). When AKT signaling is down-regulated, ROS levels are reduced due to both decreased mitochondrial activity and increased expression of antioxidant enzymes. Therefore, it can be deduced that *Bacillus* SC06-mediated inhibition of PI3K/AKT might be responsible for alleviating DQ-induced oxidative stress and apoptosis by decreasing ROS production. However, further studies are needed to confirm this hypothesis.

MAPK signaling pathways are proved to regulate autophagy during oxidative stress (Webber, 2010; Ha et al., 2014; Liu J. et al., 2015). Studies demonstrated that MAPKs trigger autophagy by stimulating the expression of several autophagy-related genes, such as *Atg9* and *Atg7* (Tang et al., 2013; Zhong et al., 2014), enhancing the conversion of LC3-I to LC3-II (Liu G.Y. et al., 2015) and regulating Bcl2/Beclin1 complex activity (Sui et al., 2014). In our study, SC06 pretreatment inhibited DQ-induced JNK phosphorylation and markedly increased p38 phosphorylation, while there was no significant difference in p-ERK1/2 levels. JNK can be easily activated by oxidative stress and promotes cell dysfunction and apoptosis (Hamdi et al., 2005; Liu and Sun, 2010). It was reported that JNK activation was involved in oxidative stress-induced reduction of insulin while suppression of JNK pathway could protect β-cells from oxidative stress (Kaneto et al., 2005). Consistently, in our study, DQ exposure dramatically increased JNK phosphorylation while *Bacillus* SC06 pretreatment could inhibit it. Furthermore, JNK induces autophagy through the disruption of Bcl2/Beclin1 complex, mainly contributing to autophagic cell death (Borsello et al., 2003; Wei et al., 2008; Park et al., 2009). Our results showed that the expression of Bcl2 and Beclin1 significantly increased, suggesting SC06 could not provoke autophagic cell death. Similar to our previous findings (Wu et al., 2017), the *Bacillus-*suppressed JNK activity may exert a protective function during oxidative stress. P38 MAPK is believed to exert dual functions in regulating autophagy. For instance, It was reported that glucose induced autophagy under starvation conditions by a p38 MAPK-dependent pathway (Moruno-Manchón et al., 2013). Another study demonstrated that p38 pathway limited the constitutive autophagy activity by reducing the maturation of autophagosomes (Corcelle et al., 2007). Noticeably, numerous studies showed that p38-induced autophagy plays a pivotal role in protecting against oxidative stress and apoptosis (He et al., 2013). Zhong et al. (2014) found that copper induced protective autophagy through transcriptional regulation of autophagy genes by activating p38 pathway in HeLa cells. P38 activated autophagy to inhibit ROS-mediated apoptosis in L929 cells (Cheng et al., 2009). Similarly, we found that p38 phosphorylation was markedly upregulated by *Bacillus* SC06 pretreatment during DQ-induced oxidative stress. Cells treated with p38 inhibitor SB203580 showed a decreased expression of LC3 and Beclin1, implying a close relationship between p38 and SC06-induced autophagy. Moreover, the apoptotic cells and ROS production were dramatically reduced with SB203580 pretreatment, which further confirmed SC06-mediated p38 activation participated in alleviating oxidative stress.

**FIGURE 12:**
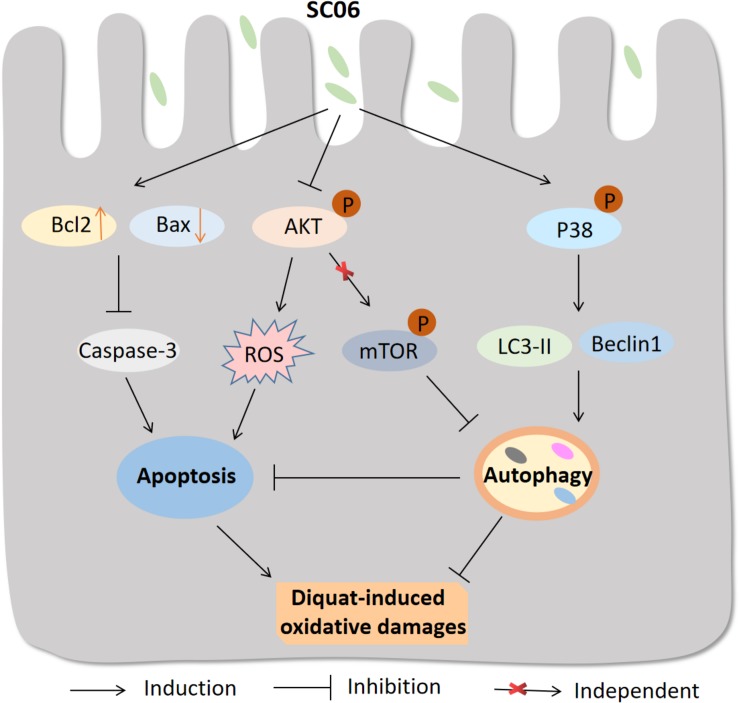
Proposed model of the protective effect of *Bacillus* SC06 in DQ-induced oxidative stress and apoptosis. (1) *Bacillus* SC06 modulates the expressions of the apoptotic proteins Bcl2, Bax and caspase-3; (2) SC06 down-regulates AKT activity to decrease ROS production; and (3) SC06 induces autophagy via the activation of p38 MAPK, not the AKT/mTOR signaling pathway. Through this mechanism, *Bacillus* SC06 protects against DQ-induced oxidative damages and apoptotic cell death.

In summary, the possible molecular mechanisms underlying the protective effects of *Bacillus* SC06 on attenuating oxidative damages are summarized as follows: (1) SC06 induces protective autophagy via the activation of p38 MAPK signaling pathway; (2) SC06 decreases apoptosis by modulating the expression of the apoptotic proteins Bcl2, Bax and caspase-3; (3) SC06 inhibits AKT phosphorylation which may contributes to ROS reduction (Figure 12). To our knowledge, this is the first report to show that *Bacillus* can directly activate host autophagy to attenuate oxidative stress-induced intestine injury. This study suggests the potential application of probiotic *Bacillus* in the prevention and treatment of oxidative stress-induced diseases and improve human or animal health.

## Data Availability Statement

The raw data supporting the conclusions of this manuscript will be made available by the authors, without undue reservation, to any qualified researcher.

## Ethics Statement

Animal studies were carried out according to the National Institute of Health Guide for the Care and Use of Laboratory Animals. All procedures were approved by the Institutional Animal Care and Use Committee of Zhejiang University (Approval Number: ZJU20160416).

## Author Contributions

WL, YPW, and YGW conceived and designed the experiments. YPW, HX, BW, LT, and LG conducted the experiments. YPW and BW analyzed the data and made the figures. YPW wrote the manuscript. WL, YGW, YL, and LT revised the manuscript. All authors reviewed and approved the final manuscript.

## Conflict of Interest

The authors declare that the research was conducted in the absence of any commercial or financial relationships that could be construed as a potential conflict of interest.

## Supplementary Material

FIGURE S1 Pictures of the gastrointestinal tract of rats after DQ exposure.
