# Supplementary figures and images for: Probiotic Bacillus Attenuates Oxidative Stress- Induced Intestinal Injury via p38-Mediated Autophagy
Source: Front Microbiol. 2019 Sep 30;10:2185. doi: 10.3389/fmicb.2019.02185 (PMC6779063; doi:10.3389/fmicb.2019.02185)

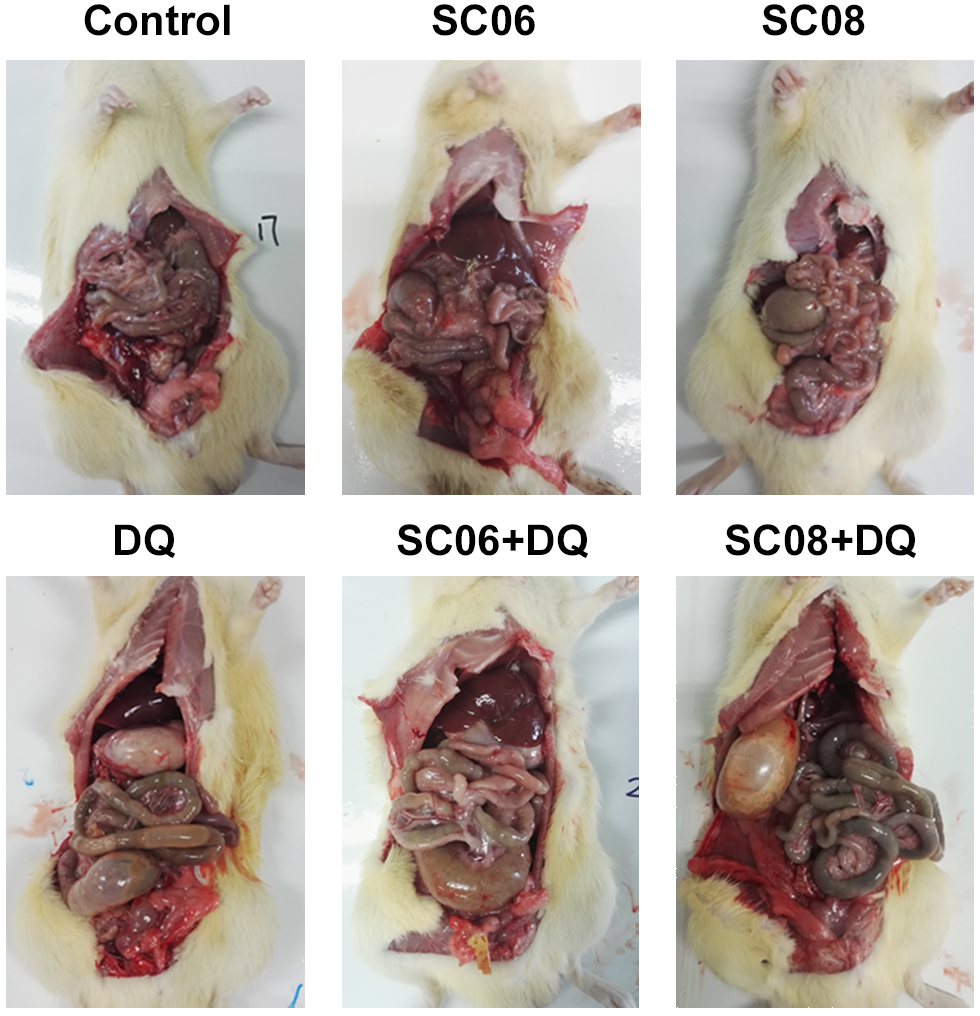

Supplement: FIGURE S1 — Pictures of the gastrointestinal tract of rats after DQ exposure. [file Image_1.TIF]
